# Quantitative interpretation of Sedia LAg Assay test results after HIV diagnosis

**DOI:** 10.1371/journal.pone.0271763

**Published:** 2022-07-28

**Authors:** Joseph B. Sempa, Eduard Grebe, Alex Welte

**Affiliations:** 1 Faculty of Health Sciences, Department of Biostatistics, University of the Free State, Bloemfontein, South Africa; 2 South African Department of Science and Technology—National Research Foundation Centre of Excellence in Epidemiological Modelling and Analysis, Stellenbosch University, Stellenbosch, South Africa; 3 Vitalant Research Institute, San Francisco, California, United States of America; Universidad Rey Juan Carlos, SPAIN

## Abstract

**Background:**

Testing for ‘recent HIV infection’ is common in surveillance, where only population-level estimates (of incidence) are reported. Typically, ‘recent infection’ is a *category*, obtained by applying a threshold on an underlying continuous biomarker from some laboratory assay(s). Interpreting the biomarker values obtained for individual subjects, as estimates of the date of infection, has obvious potential applications in the context of studies of early infection, and has also for some years attracted significant interest as an extra component of post-test counselling and treatment initiation. The applicable analyses have typically run aground on the complexity of the full biomarker growth model, which is in principle a non-linear mixed-effects model of unknown structure, the fitting of which seems infeasible from realistically obtainable data.

**Methods:**

It is known that to estimate Mean Duration of Recent Infection (MDRI) at a given value of the recent/non-recent -infection discrimination threshold, one may compress the full biomarker growth model into a relation capturing the probability of a recent test result as a function of time t since infection, given a value of assay threshold h which defines the recent/non-recent discrimination. We demonstrate that the derivative (gradient), with respect to h. of the probability of recent infection, seen as a function of both t and h, is identical to the formal likelihood relevant to Bayesian inference of the time since seroconversion, for a subject yielding an assay result h, at or close to the date of their first positive HIV test. This observation bypasses the need for fitting a complex detailed biomarker growth model. Using publicly available data from the CEPHIA collaboration, we calibrated this likelihood function for the Sedia Lag assay, and performed Bayesian inference on hypothetical infection data.

**Results:**

We demonstrate the generation of posteriors for infection date, for patients with various delays between their last negative and first positive HIV test, and a range of LAg assay results (ODn) hypothetically obtained on the date of the first positive result.

**Conclusion:**

Depending on the last-negative / first-positive interval, there is a range of ODn values that yields posteriors significantly different from the uniform prior one would be left with based merely on interval censoring. Hence, a LAg ODn obtained on the date of, or soon after, diagnosis contains potentially significant information about infection dating. It seems worth analysing other assays with meaningful dynamic range, especially tests already routinely used in primary HIV diagnosis (for example chemiluminescent assays and reader/cartridge lateral flow tests which admit objective variable line intensity readings) which have a sufficient dynamic range that corresponds to a clinically meaningful range of times-since-infection that are worth distinguishing from each other.

## Introduction

There are many reasons to want to establish timelines of infection and disease progression in patients, across a variety of diseases. In the case of HIV, which is our current application, estimating the time of infectious exposure can assist in further investigations within studies of early pathogenesis, and also support difficult conversations during post-test counselling, which touch on such sensitive matters as where and when one may have become infected, and whom one may have put at risk.

It is understood that such estimates, based on laboratory assays, will always be subject to significant uncertainty, most notably from inter subject variability of biological processes, but it seems important to formally assess what is and is not possible, given current biomarkers which provide some sort of calibratable clock for post-infection time. Numerous HIV infection biomarkers of this kind have been identified [1–4] though they are almost exclusively used for surveillance purposes. Despite their increasing use in clinical diagnosis [5], the use of infection-dating markers in individuals remains somewhat controversial–in part because of the lack of a systematic framework for the interpretation of such data.

One of the few well-known schemes for estimating the stage or timing of infection, usually called the Fiebig staging system, provides for a number of categorical stages post infectious exposure, defined by various combinations of test positivity and test negativity of assays of varying detection sensitivity during early stages of viral replication and immune system response [6]. Most of the laboratory assays used to define these ‘Fiebig Stages’ are no longer routinely available, given the rapid evolution of the diagnostic industry, and so the original estimates of the location in time, relative to infectious exposure, of these stages are of limited practical application.

Recently, the underlying logic and statistical consistency of the concept of test-discrepancy have been more carefully elucidated by Grebe et al [7], leading to a generalisation of the Fiebig staging scheme. This generalisation provides for a flexible family of test-discordant states whose temporal meaning can be made precise with widely accessible data, which has also been curated for the benefit of potential users [8]. The notion of categorical infection ‘stages’ implies estimates of infection dates which are almost uniform probability/confidence distributions within boundaries set by test dates, with offsets implied by typical ‘diagnostic delays’ of the relevant tests [7]. In other words, while the ‘edges’ are not sharp, complete knowledge of last negative and first positive tests (ideally, but not necessarily, from different tests performed on the same date) is equivalent to the estimation of an interval in which infection is almost certainly located. Beyond the not entirely perfect boundaries of these intervals, there is little information, in such a scheme, about when, within that interval, infection is more likely to have occurred, and these intervals between last negative test and first positive may be quite large.

In order to go beyond these almost ‘pure interval’ estimates, the most obvious idea is to use the dynamic range of the biomarkers to hand, and to attempt to calibrate some informative dynamic range of values of these markers, to serve as some kind of approximate clock. While, as noted above, there is now increasing use of recent infection testing in clinical contexts, this is still largely based on categorical interpretation using some threshold [9,10], the choice (and not very clearly spelled out meaning) of which is based on application to surveillance.

We adopt, according to Grebe et al 2019 [7] and Facente et al 2020 [11], the notion of ‘Estimated Date of Detectable Infection’ (EDDI), by which we mean the date at which the infection in a particular individual would first have been detectable, using a particular diagnostic algorithm being applied. (It has previously also been used to specifically imply diagnosis by a sensitive viral load assay with a diagnostic threshold of one copy per ml of blood.) We will treat this, heuristically, as if it were the infection date, and thus avoid repeated cumbersome reference to the details of the formal procedure for incorporating the notion of assay-specific ‘diagnostic delays’ [7]. In the present context, these (heuristically bluntly interval censored) estimates are in any case merely used as Bayesian priors of infection date estimates, which precede knowledge of the continuously variable recency biomarker values.

In the present work, we demonstrate a simple approach, informed by precise formal statistical arguments, to generate continuously variable infection date estimates, when a sufficiently well characterised continuously variable infection marker is available from a test performed at or around the time of first objective evidence of infection.

Our investigation is substantially inspired by our work in using markers of infection timing to support surveillance–in particular incidence estimation [12]. As we recycle not just the intuition about biomarkers developed for that purpose, but also analytical concepts, we briefly recap that:

- Incidence surveillance using markers of ‘recent infection’ is based on the heuristic that a large ‘prevalence’ of a categorically defined ‘recent infection’, among HIV positive respondents in a survey, is an indication of high incidence in a time window preceding the survey.
- This idea has been made precise by the work of Kassanjee et al 2012 [13], which defines the key concepts and analytical relationships that are required.
- A logically valid recent infection test can essentially be constructed from almost any continuous biomarker with a reasonable dynamic range post infection / initial detectability of infection.
- Whether a test for recent infection is usefully statistically informative of HIV incidence depends in considerable detail on many factors, including context, limitations of survey size, etc. but also, crucially, on the two key performance characteristics of the test, understood to be comprised of one or more assays, with rules for dichotomising the net result into *recent infection* versus *non-recent infection*):
- The *Mean Duration of Recent Infection* (MDRI) is the mean time which individuals spend exhibiting the ‘recent’ range of the biomarker(s) employed in the test for recent infection [13]. There is nominally a time cutoff, *T*, which is required to define the upper limit of ‘valid’ recent results–one of several fine points of statistical bookkeeping. The MDRI captures mainly the biology of early pathogenesis, and, in order for the recency test to be truly useful, should not have much variation between contexts.
- The False Recent Rate (FRR) is the proportion of individuals who, despite being infected for longer than the time cut off *T*, nevertheless are classified as ‘recent’ by the laboratory procedures used to define that [13]. At least for surveillance purposes, for this to be a tolerable feature of the test, the FRR should be very small (less than one percent), though it is expected to inevitably vary between contexts.

## Methods

In this analysis, we use publicly available data for the Sedia LAg assay published as part of Sempa et al 2019 [3]. From the 2424 samples in the CEPHIA Evaluation Panel amounting to 928 unique specimens we used 453 unique samples, with a wide range of times since infection, mostly infected with HIV-1 subtype B (25.6%), C (42.6%), A1 (20%), and D (8.8%). We excluded 475 unique patients because they were either on antiretroviral therapy or elite controllers.

### Laboratory procedures

The CEPHIA Evaluation Panel was tested with the Sedia™ HIV-1 Limiting Antigen Avidity EIA (LAg) assay [14]. The LAg assay is microtitre-based with the solid phase of the microtitre plate coated with a multi-subtype recombinant HIV-1 antigen. This antigen is coated in a limiting concentration to prevent crosslinking of antibody binding, making it easier to remove weakly-bound antibody. Specimen dilutions are incubated for 60 minutes and then a disassociation buffer is added for 15 minutes to remove any weakly-bound antibody. A goat anti-human, horseradish peroxidase (HRP)-conjugated IgG is added and this binds to any remaining IgG; a tetramethylbenzidine substrate is added and a colour is generated which is proportionate to the amount of HRP. An optical density (OD) is measured for each sample and then normalized by the use of a calibrator specimen. On each plate, the calibrator is tested in triplicate, with the median of the three ODs used to normalize specimen readings, producing normalized optical density (ODn) measurements. The Sedia LAg testing procedure requires that specimens producing an initial ‘screening’ OD of ≤2.0 be subjected to triplicate confirmatory testing.

### Statistical analysis

The goal of this analysis is to quantitatively interpret a specific value of the LAg assay reading (a normalised optical density, or ODn), obtained via a standard operating procedure, into an estimate of ‘time since infection’, in a Bayesian framework. For this, we need a likelihood function that expresses the probability (density) of obtaining a particular value (*X*) of ODn on the assay, given the ‘time since infection’ t, *L*(*X*|*t*). This likelihood function captures all the biological and assay dynamic information that is relevant to our estimation.

### The likelihood function

In general, a quantitative biomarker can follow a rather complex stochastic process which captures:

- fundamental dynamics of immunology and infection,
- inter-subject variability of this dynamic, and
- measurement noise.

We can formally think of assigning each individual, at the time of infection, a vector of parameters $\left(\vec{\alpha }\right)$ from a distribution $\rho (\vec{\alpha })$. The parameters $\vec{\alpha }$ determine the ‘expected’ biomarker ($\bar{X}$) progression through $\bar{X}=F(t,\vec{\alpha })$, with particular biomarker readings (*X*) subject to a measurement error (*e*) distributed according to *σ*(*e*|*F*). The likelihood (density) of observing a value *X* of the biomarker, at a time *t* post Date of Detectable Infection (DDI), in an individual assigned the biomarker dynamic parameter vector of importance for the present purposes is formally definable as

$$L(X|t,\vec{\alpha })=\sigma (X-F(t,\vec{\alpha })|F(t,\vec{\alpha }))$$

Since we don’t ever know an individual’s parameters $\vec{\alpha }$, we need to average over all possible assignments of these parameters to get just *L*(*X*|*t*), which leads to

$$L(X|t)=\int L(X|t,\vec{\alpha })\;\rho (\vec{\alpha })\;d\vec{\alpha }$$

Obtaining realistic population level biomarker progression functions *F* through the calibration of the distribution $\rho (\vec{\alpha })$, is a daunting task, and in our view, not feasible. See for example the work of Kassanjee et al which noted that this semi mechanistic approach cannot even produce stable estimates for a much simpler summary property–the ‘Mean Duration of Recent Infection’) [15].

It is also simply not feasible to locate large numbers of individuals who are each known to be infected for some precisely estimable time, and then to observe the distribution of assay results at a healthy selection for these various times–i.e., to directly fit *L*(*X*|*t*) to data.

However, we know that it feasible is to estimate ‘Mean Duration of Recent Infection’ (or MDRI) for tests such as the LAg assay used in the present investigation. These estimates have usually relied on the interpretation of a continuous biomarker into a test for ‘recent infection’ by defining ‘recent’ as: *obtaining an assay result below some chosen threshold h*.

We know from experience in terms of benchmarking [15] and concordance of different methods from different groups using different specific regression models, that a sufficiently large data set, such as the present one obtained from previous analyses by the CEPHIA collaboration [3], can be used to fit a function

$$P_{R}(t|h)$$

by which we mean the probability (not density) that, at a time *t* after infection becomes detectable, a subject exhibits a ‘recent’ result, given the assay threshold value *h*–which in our case simply means that we obtain a biomarker result less than *h*. In terms of our likelihood defined above:

$$P_{R}(t|h)=\int_{0}^{h}L(x|t)\;dx$$

By the fundamental theorem of calculus:

$$\frac{d}{dh}P_{R}(t|h)=L(h|t)$$

So, in order to obtain the required likelihood function, we need merely differentiate *P*_*R*_(*t*|*h*) *with respect to the recency threshold h*. The slight complication is that an execution of the well known fitting procedure intrinsically produces *P*_*R*_(*t*|*h*) as function of *t*, for a given value of *h*. In order to obtain the derivatives with respect to *h*, we have implemented a numerical procedure comprised of these steps:

Fitting:

- We obtained a family of *P*_*R*_(*t*|*h*) functions using a binomial regression with a logit link function, and up to cubic terms in time, which means fitting *β*_0_(*h*) to *β*_3_(*h*) to produce a maximum likelihood fit of:
-
$P_{R}(t|h)=\frac{1}{1+e^{-(\beta_{0}+\beta_{1}t+\beta_{2}t^{2}+\beta_{3}t^{3})}}$

- We stored the fitted parameters for values of LAg ODn threshold, *h*, from 0.1 to 4.0, in steps of 0.01.

*Evaluation of Likelihood L*(*X|t*):

- We evaluate *P*_*r*_(*t*|*h*) at the target value of *t*, for the six available values of *h* nearest to *X*.
- We fit *P*_*R*_(*t*|*h*) as a quadratic polynomial **in the *h* direction**.
- We evaluate the derivative, with respect to *h*, of this fitted polynomial, at *h* = *X*. This is the likelihood density!

This procedure allows fast and smooth evaluation of the likelihood densities which are required to perform the infection time inference. In some cases, because the fitting of *P*_*R*_(*t*|*h*) is inevitably prone to some fluctuations, it is possible that the numerical derivative with respect to *h* can be negative, which is unphysical. We only observed this for values of X near the top of the dynamic range of the LAg asay, *together with* values of *t* of a small number of days–i.e. situations when it is in any case clear that the likelihood should be neglibibly small, as large assay values are implausible immediately after infection. We implemented a rule of setting the likelihood to 0 whenever the directly obtained value was negative. This had no effect on the salient features of the posteriors.

### Bayesian priors

In order to demonstrate the application to infection time inference, we considered hypothetical newly diagnosed individuals with a range of intervals between a last negative and first positive diagnostic test. As previously mentioned, there are subtleties about distinguishing ‘data of infectious exposure’ from the ‘date of detectable infection’ (DDI). We interpret our inference as an EDDI, introduced above, defining ‘detectable infection’ as detectable on the testing algorithm used at the last negative and first positive result. We also assuming, in this first exercise, that there is no connection between infection risk and timing of testing, such as in a study where the timing of testing is imposed by the study protocol. Absent strong information on incidence trends, this leads to a uniform Bayesian ‘prior’ for the EDDI. In this base case, the posteriors are simply the correctly normalised (in the time direction) likelihood densities.

In practice, most contexts have patients presenting for testing at date that are to some extent self-selected. In such cases, it is far less clear how one might assign a sensible somewhat informative prior for the EDDI. Various schemes, using epidemiological context, local knowledge about testing behaviours, and additional information gleaned from the patient at the time of testing, could be proposed. This seems to us to be important, going forward, but lies beyond our present scope. We will merely show the application of a somewhat arbitrary non-uniform prior, to indicate that our code handles this case easily enough, and to demonstrate, by comparison to an otherwise equivalent set of test results, how this can affect the inference.

Statistical analysis was done using R, version 4.0.2 (R Foundation for Statistical Computing, Vienna, Austria).

## Results

**Fig 1:**
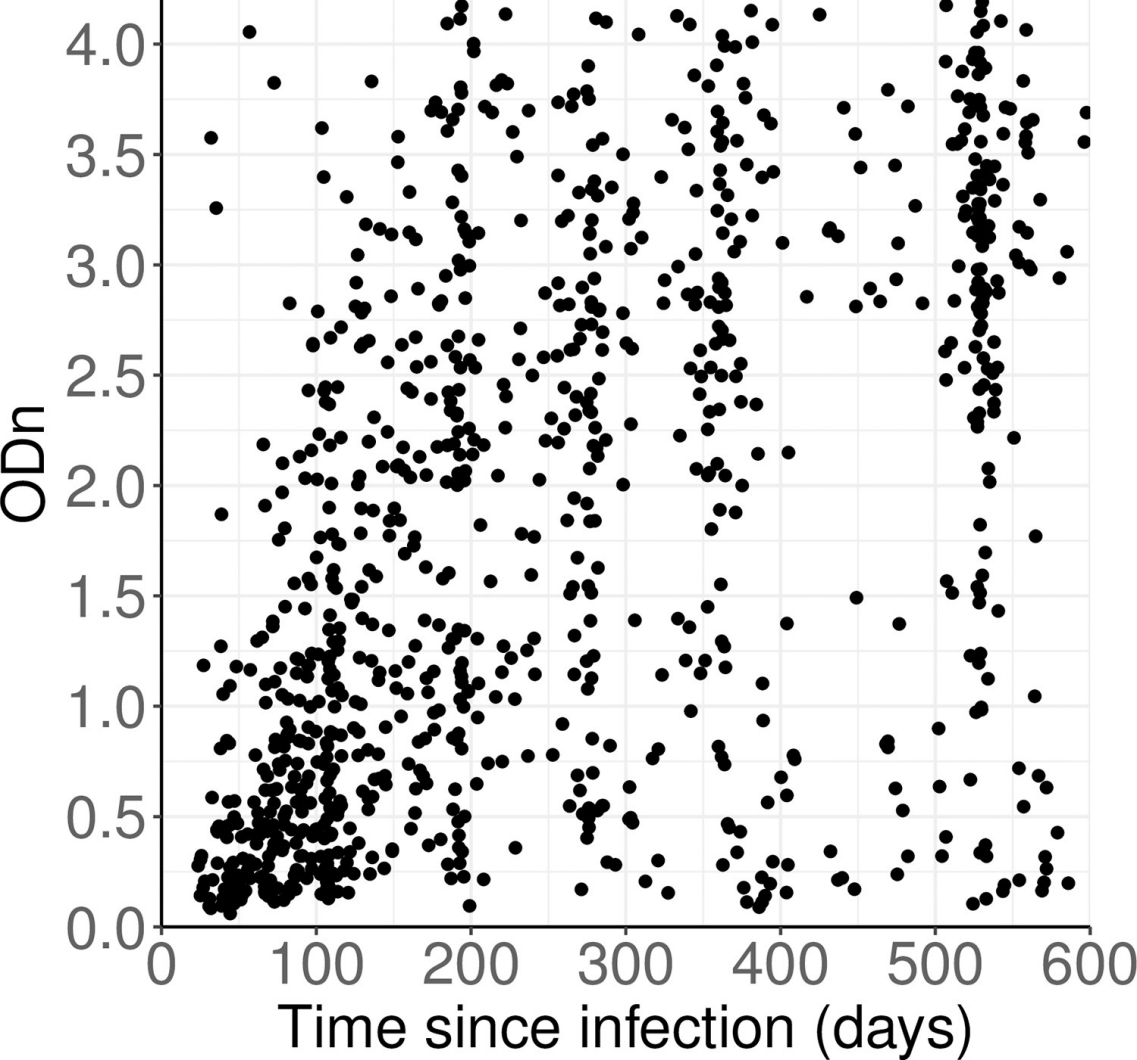
Distribution of ODn vs days since EDDI of CEPHIA evaluation panel specimens from subjects who were HIV positive, ART-naïve and non-elite controllers.

Fig 1 shows, as a scatterplot, the values of ‘time since EDDI’ and Sedia LAg Avidity normalised optical density (ODn) for the specimens in the CEPHIA evaluation panel data set which were used for the present analysis. The clustering in the time direction is not surprising and arises from the estimation of EDDI as the midpoint of the (often whole months) interval between last negative test and first positive diagnostic test.

**Fig 2:**
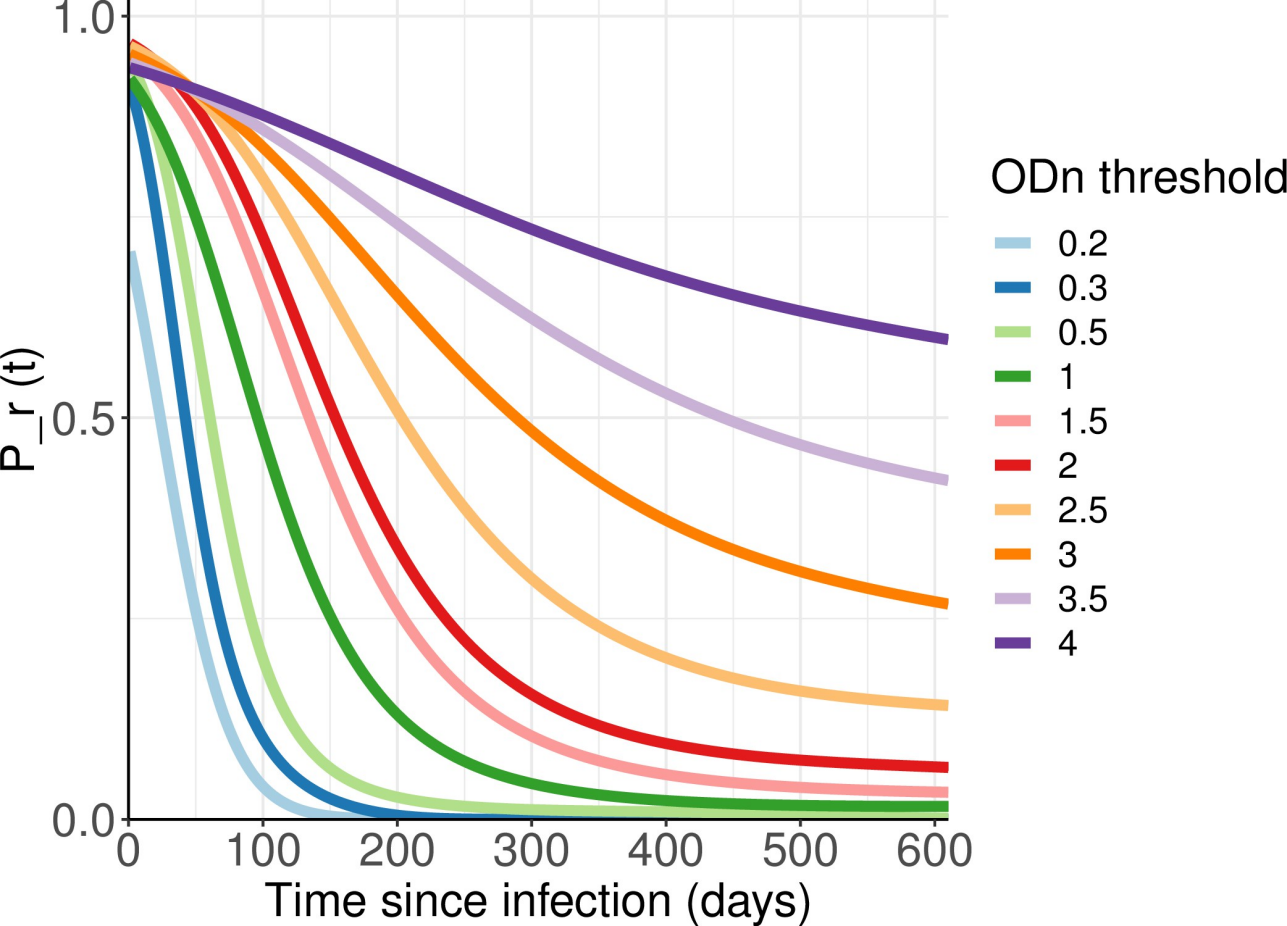
Probability of exhibiting the recent marker as a function of time since detectable infection.

Fig 2 shows *P*_*r*_(*t*|*h*) curves for the LAg assay, out to 600 days, for a range of threshold to define recent infection. At lower ‘recency’ thresholds, the *P*_*r*_(*t*|*h*) curves approach zero convincingly, while for higher ODn values (roughly ODn >2) they clearly do not approach zero.

**Fig 3:**
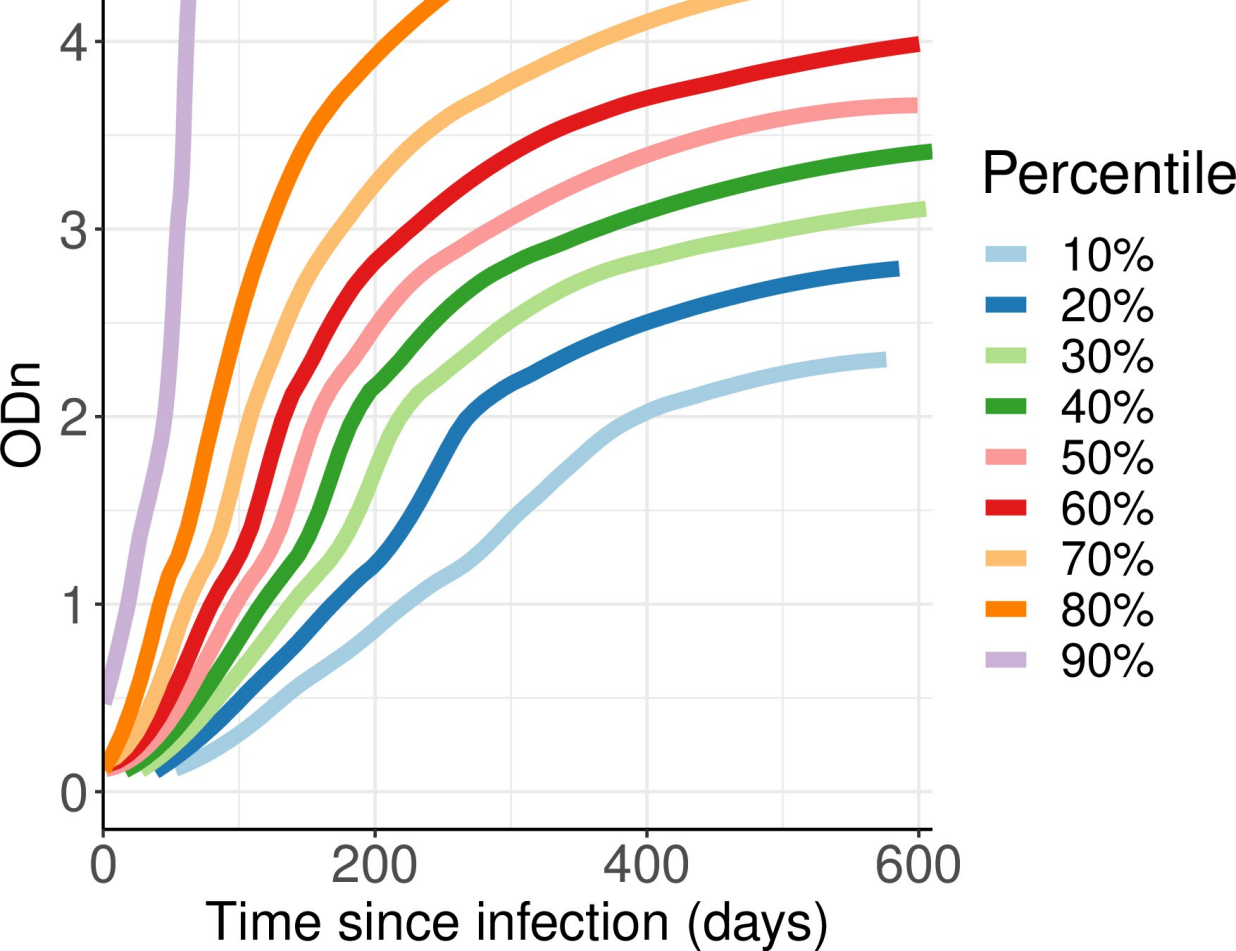
Percentile curves generated from probability of exhibiting the recent marker as a function of time since detectable infection—*P*_*r*_(*t*).

Fig 3 captures the *P*_*r*_(*t*|*h*) curves through a contour plot in deciles. This can be read much as one reads baby growth curves for height and weight, for example. Given a value of time since infection (X axis), a vertical slice through the percentile curves indicates what percentage of subjects’ ODn values lie below the Y values of the contours at the chosen X value. These growth curves are another way one might interpret/present ODn values obtained at the date of first diagnosis. However, this is still a fundamentally frequentist presentation, telling us the probabilities of seeing various values of ODn, given a time since infection, without telling us the probabilities of hypothetical values of time since infection, given an ODn. We find the Bayesian posterior approach significantly more appealing.

**Fig 4:**
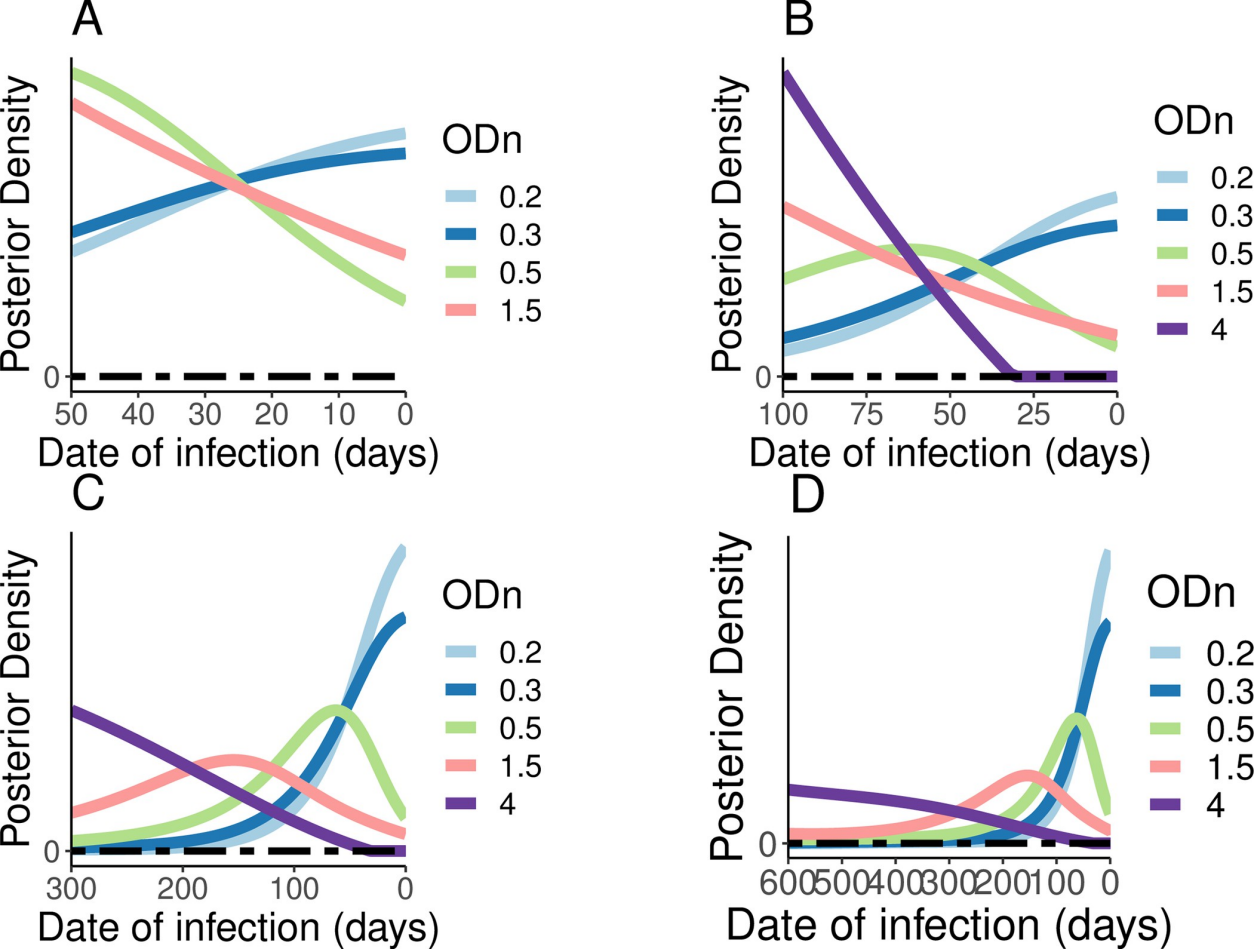
Posterior distribution of various ODn values and inter-test intervals of last negative and first positive HIV test, i.e., *L*(*h*|*t*). **A**—inter-test interval of 50 days; **B**—inter-test interval of 100 days; **C**—inter-test interval of 300 days; **D**—inter-test interval of 600 days.

**Fig 5:**
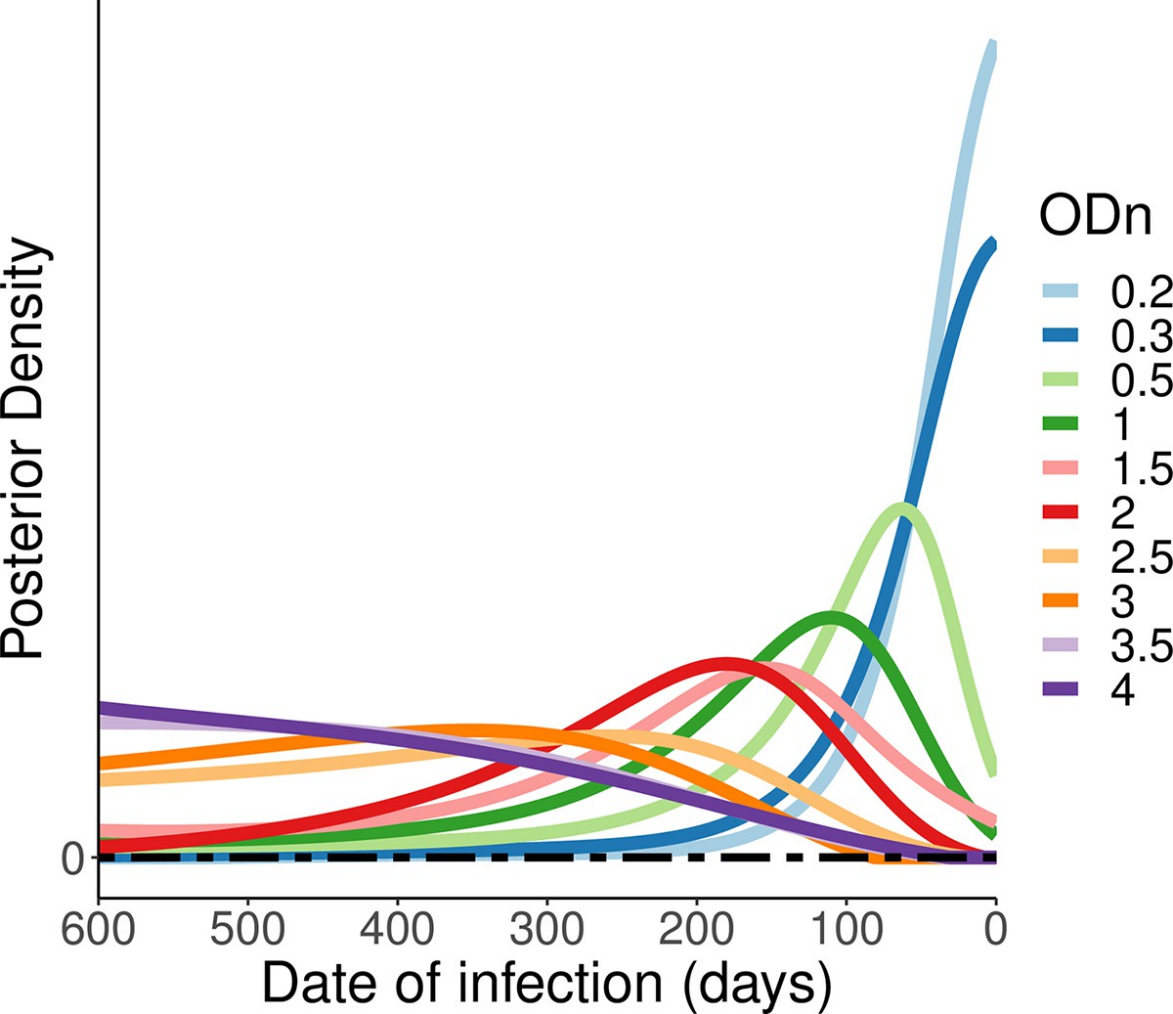
Posterior distribution of various ODn values for an inter-test interval of 600 days.

In Fig 4, we present the posterior density for a selection of values of ODn (0.2, 0.3, 0.5, 1.5 and 4) obtained on the day of first positive test, for different inter-test intervals (50, 100, 300 and 600 days), using a uniform prior. As expected, not all values of ODn are equally informative, and the value of the ODn data varies significantly with the inter-test interval. Note that in our case, where the prior distribution is always uniform, the shape of the posteriors is always the same as the likelihood itself, viewed/sliced as a function of time since infection. The families of curves look slightly different between the panels of Fig 4 because in each case we normalise the posterior sensibly to provide a cumulative probability of 1 over the inter-test interval. Fig 5 applies to the same scenario as panel D in Fig 4 but reveals a little more detail through the inclusion of a few additional values of ODn.

Note that not all posteriors have a well-defined mode inside the sensible range of values. This merely captures that for some situations there is no internal ‘most likely’ region for the infection date–it is merely increasingly likely to lie ever further to one side, within the limits sets by the last negative and first positive diagnostic tests.

**Fig 6:**
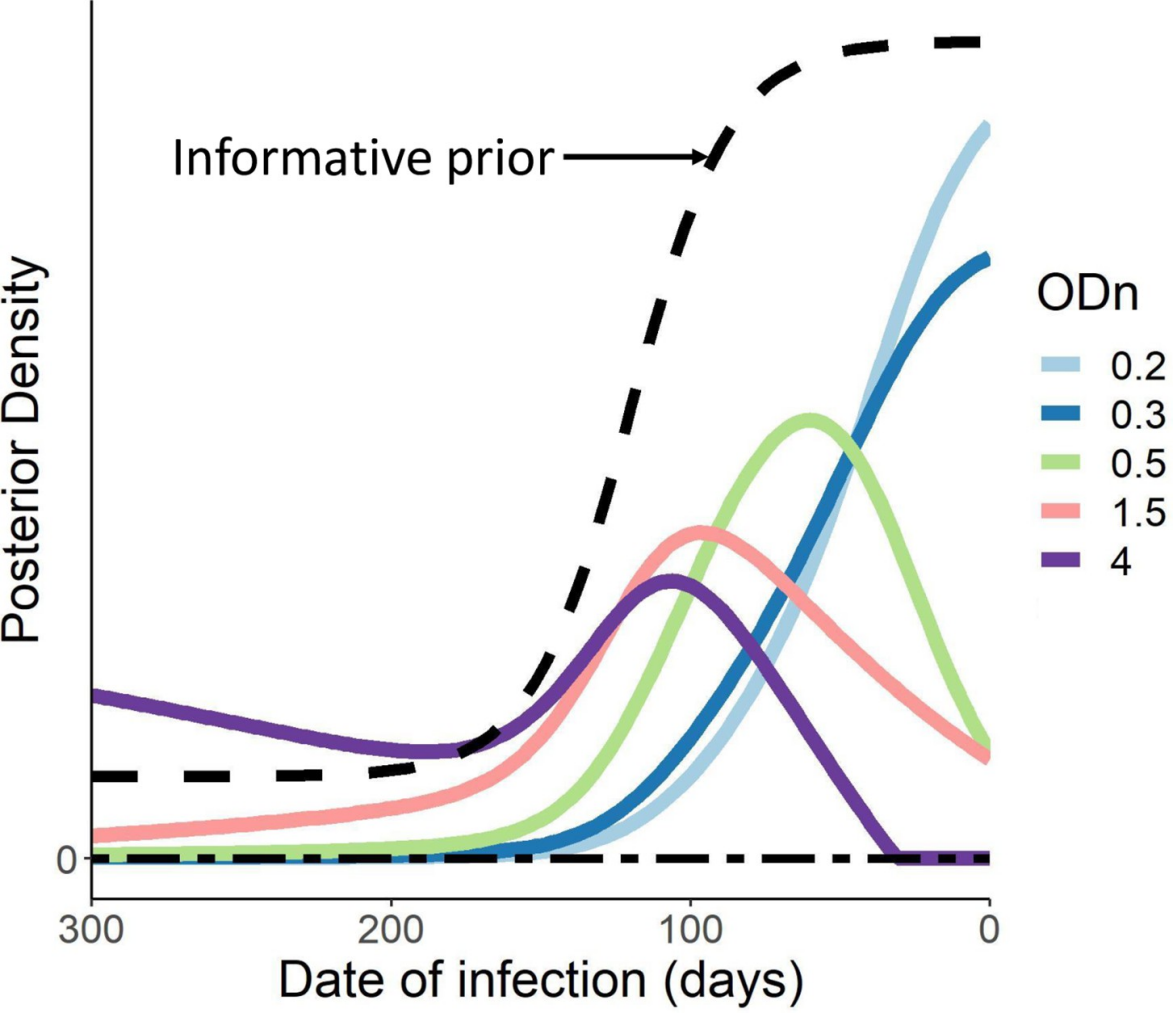
Posterior distribution of various ODn values for an inter-test interval of 300 days, using a sigmoidal prior as described in the text/indicated in Black.

To demonstrate that a uniform prior is by no means essential to our analysis, we present, in Fig 6, the posteriors for infection time, for various values of ODn obtained at the end of an inter-test interval of 300 days, and a sigmoidal prior which captures the view that risk has been greatly higher in the last 100 days.

## Discussion

Our ‘efficient’ derivation of the likelihood density, which is the crux of infection date estimation procedure, is nothing more than a usually neglected interpretation of constructs which have long been routinely used to estimate MDRI for putative recent infection tests. It does not rely on any special or controversial assumptions. Whether there is sufficient data to estimate this likelihood density without significant parametric artifacts will be a question that must be investigated for any particular biomarker intending to be used in infection timing estimates of the kind we are proposing here. For previously described markers such as the Sedia LAg avidity assay, and numerous others which have been well studied by both an original developer and an independent laboratory [1–4], it seems clear that sufficient data is available to use the proposed method outlined here for estimating infection dates.

Given a particular biomarker, we believe that the fundamental analysis developed here presents the most efficient/informative extraction of infection date posteriors that one can hope for. How useful these estimates are, in practice, will depend on a number of details which we do not claim to be investigating at the present time. In particular:

- We propose that there be some qualitative investigation into how either the ‘percentile curves’ or the ‘posterior infection date’ estimates can be used in clinical settings, to ascertain how patients and healthcare providers understand and value this information, and hence to propose meaningful guidelines for clinicians to speak about them.
- Details of biological studies and early intervention trials will determine how significantly more useful the fully developed posterior estimates are, compared to the priors obtained by the post Fiebig interval censoring of Grebe et al 2019 [7] or more informative priors of the kind we have anticipated but not constructed in any meaningful sense.

Given that the statistical ‘heavy lifting’ is done once, up front, when fitting *P*_*r*_(*t*|*h*) for various values of *h*, the computational effort involved in generating these estimates for individual patients is very modest. For further exploration, such as a possible study of utility in a clinical context, we have developed an Rshiny app (https://sempa.shinyapps.io/BM_posteriors_pctcurves/) which requests

- the last-negative / first positive test interval,
- (optionally) the specification of the diagnostic tests, or their mean ‘diagnostic delays’, and
- the specification, and value, of the recency marker obtained at or near the time of first diagnosis

and, from this information, generates the infection date posterior. Further, we are in the process of expanding it to include other recency like Maxim LAg, bio-rad Geenius, Abort Architect and some annotation like specific percentile ranges, and to cater for at least sigmoidal informative priors specified by 4 parameters (2 levels, a transition midpoint, and a transition time scale).

Despite the hesitancy in some quarters about using recent infection tests for individual level rather than population level testing [16], we note that it is increasingly being done, and will continue to be done, whether or not there is consensus on its meaning and appropriate use. We would argue that the formalisation of Bayesian infection date estimates, which we have developed here, is a crucial component of a rational discussion about such applications, and offers substantially more information than simply classifying newly diagnosed individuals as being ‘recently infected’, or ‘not recently infected’, as is currently the prevailing practice.

## Supporting information

S1 File (CSV)
